# Professional liability insurance in Obstetrics and Gynaecology: estimate of the level of knowledge about malpractice insurance policies and definition of an informative tool for the management of the professional activity

**DOI:** 10.1186/1756-0500-4-544

**Published:** 2011-12-17

**Authors:** Serena Scurria, Alessio Asmundo, Claudio Crinò, Patrizia Gualniera

**Affiliations:** 1 Departmental Section of Legal Medicine, University of Messina, Messina, Italy

## Abstract

**Background:**

In recent years, due to the increasingly hostile environment in the medical malpractice field and related lawsuits in Italy, physicians began informing themselves regarding their comprehensive medical malpractice coverage.

**Methods:**

In order to estimate the level of knowledge of medical professionals on liability insurance coverage for healthcare malpractice, a sample of 60 hospital health professionals of the obstetrics and gynaecology area of Messina (Sicily, Italy) were recluted. A survey was administered to evaluate their knowledge as to the meaning of professional liability insurance coverage but above all on the most frequent policy forms ("*loss occurrence*", "*claims made*" and "*I-II risk*"). Professionals were classified according to age and professional title and descriptive statistics were calculated for all the professional groups and answers.

**Results:**

Most of the surveyed professionals were unaware or had very bad knowledge of the professional liability insurance coverage negotiated by the general manager, so most of the personnel believed it useful to subscribe individual "private" policies. Several subjects declared they were aware of the possibility of obtaining an extended coverage for gross negligence and substantially all the surveyed had never seen the *loss occurrence *and *claims made *form of the policy. Moreover, the sample was practically unaware of the related issues about insurance coverage for damages related to breaches on informed consent. The results revealed the relative lack of knowledge--among the operators in the field of obstetrics and gynaecology--of the effective coverage provided by the policies signed by the hospital managers for damages in medical malpractice. The authors thus proposed a useful information tool to help professionals working in obstetrics and gynaecology regarding aspects of insurance coverage provided on the basis of Italian civil law.

**Conclusion:**

Italy must introduce a compulsory insurance system which could absorb, through a mechanism of "distribution of risk", the malpractice litigation and its costs. This will provide compensation in accidental cases where it wouldn't be possible to demonstrate carelessness, imprudence and/or lack of skill.

## Background

Clinical activities in obstetrics and gynaecology have peculiar features due to the different health care providers involved. The physician specialized in obstetrics and gynaecology, the graduated nurse and nurse-midwife (obstetric nurse) have poorly defined areas of skill-competence according to each Undergraduate Degree (for nurses and nurse-midwives) and Specialisation Degree (for physicians) as well as in their pertinent "professional profiles" and respective "deontological codes".

These activities also involve "third parties" such as the father (Court of Cassation, 29/07/2004, n. 14488; Court of Cassation, United Sections 11/01/2008, n. 577) of the unborn (and the newborn) baby, who might be upset and interested in pursuing a lawsuit against the physician, nurse, and/or hospital for bad outcomes (especially in obstetrics) related to health care service. Consequently we can call this kind of activity, "triple risk activity".

Owing to the lack of specific knowledge about insurance coverage of professional liability in this medical discipline, together with some recent contradictory decisions of the Courts(Court of Bologna, 2/10/2002; Court of Crotone, 8/11/2004; Court of Cassation, III Civil Section, 15/03/2005, n. 5624; Court of Rome, 12/09/2007, Court of Genoa, 8/04/2008), we deemed it useful to examine the real coverage provided by insurance plans and the level of knowledge about malpractice insurance policies. The aim is to create a tool for the management of the professional activity.

## Methods

A closed-response based questionnaire designed to assess the knowledge and perceptions of health care professionals with regards to various types of policies ("*loss occurrence*," "*claims made*" and "*I-II risk*") and services covered by the insurance, was administered to a sample of 60 health professionals of the obstetrics and gynecology hospital area of Messina (Sicily, Italy).

The study was not submitted to ethical approval because it didn't involved patients according to Italian law.

The questionnaire included seven questions:

The signing of individual policy contracts and the level of knowledge about them among the different professional categories;

The degree of awareness of the guarantees provided by the hospital policies;

The knowledge of the possibility of warranty extension for gross (fault) negligence;

The knowledge of different types of policy (*Loss occurence, Claims made, First or Second risk)*

The knowledge of coverage provided by the contracts in cases of damage resulting from a "vitiated" informed consent.

Professionals were grouped according to the institutional role and function, strictly age-related. The age group up to 30 included physicians specializing in obstetrics and gynecology, while in the age group between 50 and 60 years old included obstetricians-gynecologists and graduated nurse and nurse-midwife (obstetric nurse).

Descriptive statistics were calculated for all the groups (categories) of health professionals and answers to the questions (YES/NO/I DON'T KNOW). A *χ*^2 ^test was conducted to explore the relationship between the respondent's level of knowledge about malpractice insurance policies and each group (category) of health professionals involved: obstetrics/gynecologist, obstetric nurse, physician-surgeon in residence. The level of significance was set a *P*-value of 0.05.

## Results and discussions

The surveys (Table 1) show very poor knowledge of the coverage provided by the insurance policies subscribed by the hospitals. Thirty-three percent (33%) of the surveyed professionals were unaware while 27% had a very bad knowledge of the professional liability insurance coverage subscribed by the general manager. We found that medical specialists had a better knowledge of the insurance coverage in their contract. This is confirmed by a statistical significance (*p *0.02 0.02).

**Table 1:** Knowledge of the professional liability insurance coverage in a sample of hospital health professionals (*n *= 60)

	Knowledge of private policies						
	**None**	**Poor**	**Middle**	**Good**	**Total**		

Obstetricians-gynecologists	11	3	10	10	34	*χ*^2^	5,979

Obstetric nurse	7	1	2	4	14	Degree of freedom	6

Physician-Surgeons in residence	8	0	1	3	12	*P*-value	0,201

	Knowledge of hospital policies						

	None	Poor	Middle	Good	Total		

Obstetricians-gynecologists	8	8	15	3	34	*χ*^2^	11,364

Obstetric nurse	9	3	2	0	14	Degree of freedom	6

Physician-Surgeons in residence	3	5	4	0	12	*P*-value	0,023

	Signing of individual policy						

	Yes	No	Total				

Obstetricians-gynecologists	23	11	34			*χ*^2^	4,583

Obstetric nurse	7	7	14			Degree of freedom	2

Physician-Surgeons in residence	4	8	12			*P*-value	0,101

	Warranty extension for gross negligence						

	Yes	No	Total				

Obstetricians-gynecologists	32	2	34			*χ*^2^	17,501

Obstetric nurse	6	8	14			Degree of freedom	2

Physician-Surgeons in residence	6	6	12			*P*-value	0,001

	Knowledge Loss/Claims						

	Yes	No	Total				

Obstetricians-gynecologists	6	28	34			*χ*^2^	5,098

Obstetric nurse	0	14	14			Degree of freedom	2

Physician-Surgeons in residence	0	12	12			*P*-value	0,078

	Knowledge first/second risk						

	Yes	No	Total				

Obstetricians-gynecologists	18	16	34			*χ*^2^	13,579

Obstetric nurse	1	13	14			Degree of freedom	2

Physician-Surgeons in residence	1	11	12			*P*-value	0,001

	Knowledge warranty for vitiated informed consent						

	Yes	No	I don't know				

Obstetricians-gynecologists	7	18	9			*χ*^2^	9,197

Obstetric nurse	0	4	10			Degree of freedom	4

Physician-Surgeons in residence	2	5	5			*P*-value	0,056

Fifty-seven percent (57%) of the gynaecology and obstetrics staff had time contracts. The latter seemed to have a good knowledge, while physicians or surgeons in residence had less knowledge as to the effective coverage offered by the hospital general liability policy. We found no significant relationship between the two groups.

Seventy-three percent (73%) of the professionals claimed to know about coverage extension for gross negligence damages. Ninety percent of responders (90%) had almost no knowledge of the different insurance policies available for medical malpractice (loss occurrence or claims made). Moreover, we observed a statistically significant (*p *< 0,001) frequency of extended coverage policy subscriptions for damage due to gross negligence in the group of obstetrician-gynecologists compared to nurses and nurse-midwife. Poor knowledge of "Loss/Claims" policies did not highlight statistically significant differences in the sample (*p *= 0.08). The study also showed that the obstetricians-gynecologists group had an excellent knowledge (*p *< 0.001) of "first-second risk" type policies.

Bearing in mind that there are not peculiar characteristics of informed consent in the specific obstetric field, when asked about coverage provided for damage caused by "vitiated" informed consent, 44% of the responders answered "I don't know", while 17.3% believed to be "covered" for that risk. These results highlight poor knowledge about the argument considering that procedures concerning the informed consent are nearly always submitted to such practitioners. We found no significant relationship between the groups and the knowledge about the coverage for damages related to breaches on informed consent (*p *= 0.05).

These results allow us to state that the obstetrics-gynecology professionals have no knowledge (or very little knowledge) of the real coverage provided by hospital's medical liability insurance policies. This is mainly due to the fact that professionals are not personally involved in the drawing up of the insurance contract.

In some cases it was impossible to determine if a better level of knowledge was provided by practitioners who had previously activated insurance procedures because sued for malpractice.

Our results, however, demonstrate that obstetric/gynaecologist have a better knowledge of hospital policies and first and second risk policies with respect to other categories. This is confirmed by fact that the obstetric/gynaecologist more frequently draw up coverage extension for gross negligence.

Based on these results, we deem that an instructive tool is very useful for management of the professional activity. Given the growing number of cases of medical malpractice in Italy [1], especially in the obstetrics and gynecology field, it is necessary for every practitioner to compare information on professional liability insurance coverage of such policies, as well as best premiums in order to get the best possible deal. We believe it is also important to begin with the juridical classification of such insurance contracts.

The Italian Civil Code (c.c.), Book Four (*Obligations*), Title Three (*Specific Contracts*), Chapter 20 (*Insurance*), Section One (*General Provisions*), Article 1882 ("*Notion*"), provides that "*Insurance is the contract whereby the insurer, on the payment of a premium, binds himself to compensate the insured, within the limits agreed upon, for damage caused to the insured by an accident, or to pay a principal sum or an annuity upon the happening of an event contingent upon human life*".

Such agreement should be in a written form according to the article 1888 c.c. ("*Evidence of the contract. A contract of insurance shall be evidence in writing*"). The insurer is bound to deliver to the contracting party the insurance policy or other document signed by him. The insurer is also bound to deliver, upon request and at the expense of the contracting party, duplicates or copies of the policy; but in such case the insurer can ask that the original be shown or returned. The requisite contract or document is not the "slip" signed by the underwriter(s), but the Client's document. Although such contract need not be signed by the Client to be valid, it is strongly recommended that the Client's signature be included. For the purposes of the provisions of arts. 1892-1893 of the C.C., each Insured hereby declares that he has not received any claim for compensation with regard to culpable conduct, nor is he aware of any element that might lead to the assumption that the obligation to provide compensation might arise, as early as at the time of conclusion of the contract, on account of a deed ascribable to him which just constitutes its evidence ("*ad probationem*") because submitted (signed) by the parties. The insurer must then release the policy form or document signed by the contracting party.

According to the article 1899 c.c. *("Duration of insurance. Insurance is effective from midnight of the day on which the contract was entered to midnight of the last day of the period stipulated in the contract. If the duration of the contract exceed 10 years, the parties, after this period and regardless of agreements to the contrary, can withdraw from the contract through a 6 month advance notice, which can also by given by means of a registered letter. The contract can be tacitly extended one more time, but for a duration that cannot exceed 2 years. The provision of this article do not apply to life insurance")*, the duration of the policy--up to the last suitable contractually established day--is decided by the parties and then selected by the Contractor/Insured and indicated in the Policy Form.

Contract is drawn-up on the basis of declarations of the contracting parties and imprecise declarations or reticence of the Insured due to circumstances that have an influence on risk evaluation according to article 1892 c.c. ("*Misrepresentations or fraudulent or grossly negligent failure to disclose. If the contracting party, fraudulently or through gross negligence, misrepresents or fails to disclose circumstances which, if known to the insurer, would have caused him to withhold his consent to the contract, or to withhold his consent at the same conditions, the insurer can annul the contract*"), article 1893 c.c. ("*False representation of withholding of information without fraud or gross negligence. If the contracting party has acted without fraud or gross negligence, misrepresentation or failure to disclose are not grounds for annulment of the contract but the insurer can withdraw from the contract by means of a declaration to be made to the insured within 3 months from the day on which the insurer had knowledge of the falsity of the representation of the failure to disclose. If the accident occurs before the insurer has knowledge of the falsity of the representation of the failure to disclose, or before he has notified the insured of his intention to withdraw from the contract, the amount due by him is reduced in proportion to the difference between the premium agreed upon and the premium which would have been applied had the true situation been known*"), and article 1894 c.c. ("*Insurance in name of or on behalf of third persons. In the case of insurance subscribed in the name of or on behalf of third persons, if such persons had knowledge of the falsity of the representations or of the failure to disclose information relating the risk, the provisions of Articles 1892 and 1893 apply in favour of the insurer*") may incur the total or partial loss of the right to assistance and the termination of the insurance. Otherwise, for the purposes of the provisions of articles 1892-1893 c.c., each Insured hereby declares that he has not received any claim for compensation with regard to culpable conduct, nor is he aware of any element that might lead to the assumption that the obligation to provide compensation might arise, as early as at the time of conclusion of the contract, on behalf of a deed imputable to him.

Article 1917 c.c. deals with the legal situation of civil "*Liability insurance*", ruling that: *"In liability insurance the insurer is bound to compensate the insured for the damages which the latter must pay to a third person because of events occurring during the insurance period and resulting in the liability referred to in the insurance contract. Damages deriving from fraudulent acts are excluded.[...]"*. Thus, the contract object is the casualty of the Insured (negligence or failure to meet the acceptable standard of care owed to the patient) that caused injury to third party.

Two types of contracts are derived from this model, the so-called "*Loss occurrence*" and "*Claims-made*."

The first of these "**Occurrence**" policies provide coverage for insured events occurring during the policy period, regardless of the length of time that passes before the insurance company is notified of the claim. This is generally considered the broadest form of coverage. It is also usually the riskiest for the insurer and the most expensive for the policyholder. In fact, occurrence policies often, especially in the last few years, are not offered for medical malpractice policies because claims may be reported years after the underlying policy has expired. It is very difficult for insurers to estimate the eventual number and cost of those claims in the years following expiry.

The more recently applied "**Claims-Made**" policies provide coverage for insured events occurring during or after the specified policy's retroactive date; when the insured events are *reported *during the policy period. If the retroactive date is the beginning of the policy period, the policy is relatively inexpensive and is called "first-year" claims-made. However, as the number of years from the retroactive date increases, the policy "matures," and the premiums increase each year using "step factors" until they reach the mature level (about 5-8 years after the policy's retroactive date). Once the mature level is reached, the premium approaches the occurrence premium. Claims-made policies are the most widely available form of medical malpractice coverage today and can vary between insurance carriers, depending on the definition of a reported claim.

At the expiration of an insured's final claims-made policy, it is necessary to obtain coverage for any latent, as-yet unreported claims that may exist as a result of past medical incidents. This coverage closes the gap between claims-made and occurrence coverage. In many cases, these claims can be covered by purchasing "*Extended Reporting Period*" ("*ERP*" or "*Tail*") Coverage from the insurer at the time of policy expiration. If the insured will continue practicing and is only changing insurers, it may also be possible to obtain "*Prior Acts*" (or "*Nose*") Coverage from a subsequent insurer.

The *Modified Occurrence Policies *combine aspects of claims-made and occurrence policies.

Coverage is provided on a claims-made basis with an included ERP. The ERP generally applies for a limited time after expiration of the last policy issued. This is usually a period of 7 years. At the end of the included ERP, the insured may then be given the option of buying an unlimited ERP.

The claims-made-form seems to be contrary to the provisions of article 1917 c.c. (as it would exclude from insurance coverage the damage incurred--but not claimed--during the period that the policy is active, thus leading to a limitation of the Insurer's liability).

The only apparent conflict lies on the meaning attributed to the term "*events occurring during the insurance period*" used in the article 1917 c.c. In fact, professional liability, the same term could be represented by the negligent error or the injurious event (resulting damage or so-called "wrongful act") or the claim. However, there may be a particularly long period of time between the negligent error and the activation of insurance coverage.

The Italian Supreme Court of Cassation in the already cited judgement n.5624 of 15.3.2005 has identified the term "*event occurring during the insurance period*" with the "*claim*", just interpreting the article 1917 c.c. through the comma three of the article 2952 c.c. in Book Six ("*Protection of Rights*"), Title Five ("*Prescription and Forfeiture*"): "*Validity periods. Rights deriving from the contract are limited to a period of 1 year from the date on which such right is proved founded*", ***("With regard to third party liability, the validity period begins from the time in which the injured party submits a claim for settlement to the Insured". EIMA international.)***, which provides that the limitation period runs from the claim. In fact, the 5624/05 judgement of the Supreme Court provides that *"... the introduction of the Claims Made insurance system has done nothing else than transpose--as in a "contractualization"--a correct interpretation of the rules of the Civil Code concerning liability insurance. [...] In conclusion, the following principle of law should enunciate: liability insurance contract in the Claims made form doesn't submit the typical abstract case provided by article 1917, but is an atypical contract, generally considered lawful according to article 1322 c.c. (.. Parties are free to determine the content of the contract within the limits imposed by law. Parties may also conclude contracts that do not belong to types having a particular juridical regime, provided that such contracts are designed to realize interests worthy of protection by the legal order*"). However, the parties' right to freely determine the terms of the contract within the limits set by the law is recognised as being part of the principle of freedom of contract. It does not therefore preclude the parties to a contract from deciding to insert therein a termination clause which is not subject to the condition that the contractor must be responsible for non-performance, through the derogation from the usual format of contracts under Italian law (Judgment of the European Court, Fifth Chamber, of 27 April 1999, Commission of the European Communities, Case C-69/97, European Court reports 1999 Page I-02363) [2].

On this issue the Court of Genoa, Section Two, recently ruled in a different way (Judgment 8.4.2008) identifying the claims made policy as an atypical contract, null and void as an act that is contrary to the inflexible rule stated by the article 1917 c.c. and for lack of cause. The decision highlighted that the purpose of the insurance contract, according to the literal meaning of article 1917 c.c., is to transfer the risk resulting from professional activity (from the insured to the insurer). In other words, the insured bears a risk for some kind of loss; and pays the premium to the insurer in exchange for the insurer's promise to accept the risk. However, the claims made form of insurance is characterized by the lack of just that risk transfer--through insurance--to the insurer ("... *The insured object must be the risky activity, not the claim*"). *Claims made *policy must not be considered an "*insurance contract*". More recently the Court of Milan (Judgment 18.3.2010, n.3527) states that *claims made *policy are not an atypical contract--as ruled instead by the Court of Cassation in 2005--and that *claims made *clause is valid because it allows to satisfy the interest of the contractual parts. "Claims made" form of policy can be used to provide retroactive protection against medical errors, or to provide coverage for incidents that have occurred during the "active policy period", but only claims which are first made against the insured and reported to the Insurance Company while the policy was active or during any applicable extended reporting period ("tail coverage"). For this reason a claims-made policies expose the insured to risk not being covered for a claim discovered after the policy has expired. A good policy ensures that the policyholder is covered for professional liability even after the practitioner has retired or leaves a healthcare setup for another. The coverage for the time when the practitioner is np longer working in the place where negligence was alleged can be bought as either "Tail Coverage" or "Nose Coverage". While tail coverage is the insurance coverage provided for the previous workplace, "Nose Coverage" is the insurance cover for the new workplace subscribed in advance. Therefore, if the policyholder decides to terminate a claims-made policy, he will need to purchase "Tail Coverage" in order to continue to protect himself. This will extend the time that a claim can be reported, but the incident would still need to occur while the policy was active, or the (presumed) insured will not be covered. These aspects are strictly connected to the risk correlated to the succession of insurance contracts made with several Insurance Companies. A claim related to an event occurring before the expiry of the new contract could not find adequate coverage without retroactive protection ("Nose coverage") in the later policy [3].

According to the Italian law, as well as in Malpractice Litigation, "*Fault*" of a subject (as the conscious behaviour of a person not intentionally oriented to cause a damage, but acting with "***negligence, imprudence, technical incompetence, or inobservance of laws, rules, orders, discipline***": article 43 of the Italian Penal Code), "*harm*" to the patient (i.e. onset or prolongation of illness, permanent injury, or death) and "*causation*" (articles 40-41 of the Italian Penal Code) must be proven. Furthermore, according to the **Judgment of the Constitutional Court n.166 dated 28.11.1973 **"... ***while for the first (technical incompetence) the indulgence of the judgment is directly proportionate to the difficulties of the assignment, for the other two forms of fault (negligence and imprudence) every judgment must be conducted according to criteria of normal severity***". That's why Negligence, together with Imprudence and/or Incompetence attributable to a professional misconduct with an average skill level lower than that ordinarily required in carrying out the professional activity, in Italy is referred to as "***Gross Fault***." A particular item of the insurance coverage offered by professional liability policies concerns the intentional and gross (fault) negligence injuries produced by the insured. In fact, as provided in the article 1900 c.c. ("*Accidents caused by fraud or gross negligence of insured or persons for whose acts insured is answerable. The insurer is not liable for accidents caused by fraud or gross negligence of the contracting party, by the insured, or by the beneficiary, unless there is an agreement to the contrary for cases of gross negligence. The insurer is liable for accidents caused by the fraud or gross negligence of persons for whose acts the insured is answerable*"), the insurer is not obliged to provide coverage for casualties (harm and injuries) related to intentional, criminal or reckless misconduct or gross negligence of the contractor, the insured, or the beneficiary (the *"persons for whose acts the insured is answerable"*), unless there is a contrary agreement just for gross negligence. In fact, most professional liability insurance contracts provide for a special extension of policy to cover loss caused by gross negligence. Thus, to begin with the insurer covers any peril that is not specifically excluded ("*all risk coverage*"), however the policyholder will have to pay an additional premium so the insurance company disclaiming the insured for damages caused by (gross) negligence (or intentional misconduct).

With regard to warranty exclusions, the insurance coverage is actually less restrictive than in the past. It includes damages caused for ex. by blood transfusion or blood derivatives and related to "*collection, storage and distribution of human blood, blood components and plasma derivatives*". Insurance protection is still denied for damages resulting from aesthetic and physiognomic treatments if they are not carried out for reconstructive purposes (injury, illness or functional malformation). However, if medico-legal evaluation should confirm the necessity since aesthetic treatments could be triggered by psychological pain together within a mental illness or disorder. In fact, aesthetic surgery is usually not covered by health insurance because it is "elective" and practiced on demand, without a real health problem. Reconstructive surgery is performed on abnormal structures of the body caused by congenital defects, developmental abnormalities, trauma, infection, tumors or disease. It is generally performed to improve function, but may also be done to approximate a normal appearance. Reconstructive surgery is generally covered by most health insurance policies although coverage for specific procedures and levels of coverage may vary greatly.

Insurance Companies also offer the so-called "*second risk*" products, as contracts usually awarded to professionals which already receive insurance coverage in order to be insured in excess to the *Maximum Sum Insurable *provided by a "*first risk*" policy.

We have observed that in all the inspected insurance contracts nothing is explicitly stated about the real protection provided by hospital's medical liability insurance policies for damages related to breaches on informed consent and/or incorrect-incomplete compilation of clinical documents.

However, given the definition of the object of insurance in such contracts, referring to the "***unintentional damage caused to third parties for death, personal injury and damage to things, as a result of an accident that come about due to the activities carried out, including all operations and subsidiary and/or complementary ancillary activities...***," we couldn't exclude the coverage for biological damages resulting from any breach (including omissions) of these activities which are strictly inherent to professional services contractually due by the hospital (general manager) and hospital workers (physicians and surgeons, dentists, nurses, midwives, physical therapists, radiologic technologists and technicians, etc.).

Moreover that coverage is specifically provided with appropriate contractual clauses in some individual insurance contracts.

The insurance would be rather ineffective in cases of "vitiated" informed consent when the breach does not impact at all on physical and psychological integrity of the patient, but only coercing the "constitutionally protected" patient's right to self-determination (Constitution of Italian Republic, Part I (*Rights and Duties of Citizens*), Title I (*Civil Relations*), Article 13: "*Personal liberty is inviolable. No one may be detained, inspected, or searched nor otherwise subjected to any restriction of personal liberty except by order of the Judiciary stating a reason and only in such cases and in such manner as provided by the law*"). In that case the compensation for a "*non-pecuniary damage*" will be directly sustained by the insured (about the most recent definition of "*non-pecuniary damage*" see Court of Cassation, United Civil Sections 11/10/2008, n. 26972 and regarding the *non-pecuniary damage *related to a breach in the procedure concerning the informed consent see Court of Venice 4/10/2004, Court of Milan 29/03/2005 n. 3520, Court of Varese 20/2/2006 and Court of Cassation 14/3/2006, n. 5444[4]).

Finally, it seems appropriate to point out that during the last decades Italian Legislation has introduced some rules (Legislative Decree 19/6/1999, n. 229 "*Rules for the rationalization of the National Health System*, according to the article 1 of the Law 30/11/1998, n. 419") about the possibility to practice private professional activity inside public hospitals as "*intramoenia professional activity*," thus triggering the need to reshape of the insurance contracts in order to provide coverage for damages derived to third parties by professional (and private) activities which are assimilated to those performed as public employee within the hospital.

In fact, almost all insurance policies underwritten after the introduction of the legislation ruling the private professional activity within public hospitals, include the clause extending insurance coverage for damage produced by health professionals during "intramoenia activity."

Professional practice during post-graduate residency formation courses ("Specialisation Schools"), is protected as that carried out by workers without an employment status who participate in hospital activities. These physicians and surgeons attending specialistic training courses have a specific professional-formation contract, which means they have "bounded autonomy" provided by law (**D. Lgs. 17.8.1999, n.368 **(Enforcement of Council Directive 93/16/EEC of 5 April 1993 to facilitate the free movement of doctors and the mutual recognition of their diplomas, certificates and other evidence of formal qualifications), **Article 38**: "***The training of the medical specialist includes the assisted share to all the medical activities of the Functional Unity to which he is assigned by the School Council, as well as the gradual assumption of care assignments and carrying out of operations with an autonomy bounded to the tutor directives"***).

The conclusion of a typical employment contract for doctors attending at training courses should include the same treatment provided for registered medical staff, thus allowing them to join the extended insurance coverage for damage committed by "gross fault". In fact, most of the policies provide the insurer's duty to pay the indemnity if the insured event has been caused by the insured, policyholder, beneficiary or third person committing "ordinary negligence" and if it does not contradict the insurance contract (the extended coverage for "gross fault" will oblige the policyholder-insured to pay an additional premium) and however avoiding the possibility to ***redraft ***by the Insurance Company.

Furthermore, insurance coverage of health care workers in various disciplines not only for obstetricians and gynecologists, is the focus of a renewed interest, in our country as in the U.S.A. [5]. The ***Italian National Federation of Colleges of Medical Doctors and Dentists ***(***FNOMCeO***) [6] in April 2008 appointed a Commission to develop a reform plan for the improvement of the *current *professional (medical) *liability *system.

The result was a Draft to establish new standards for professional liability according to the Penal Code. Informed consent, has revisited the matter of civil liability of health care practitioners. A system based on the principle of strict liability of hospitals (in connection with the equipment and supplies furnished to physicians and patients) on one side and mandatory insurance coverage for risks (of loss or damage) arising from the insured professional activities, on the other.

Article 1 of the Draft provides a definition "***civil liability of health care structures***" for damages occurred within public or private hospitals produced by an illicit act committed by health care professionals, unless they were able to prove that all reasonable steps to prevent damages were taken. This is absolutely coherent with the evolution of jurisprudence on allocation of the burden of proof (among others, see the afore mentioned Supreme Court, United Sections, No. 577 of 11/1/2008.)

Article 2 of the Draft stipulates that, in analogy with the ***mandatory auto liability insurance ***(Law 24/12/1969 n.990 and Decree Law 23/12/1976 n.857, ratified with amendments by Law 26/12/1977 n.39), health care structures must conclude insurance contracts to cover the aforementioned risks. The Insurance Companies cannot deny the asked protection with pre-defined conditions and agreements in a specific "framework contract."

There is also the possibility for the victim to directly sue the Insurance Company (Article 3). In order to streamline the procedures currently required by the rules concerning damage claims, (Article 5) provides the institution of the "***Compulsory preliminary attempt of conciliation***" that--if successfully completed--would preclude the possibility to bring an action, otherwise determining its automatic remission if already proposed.

The document also deals with the establishment of a "**Guarantee Fund for victims of in-hospital iatrogenic injuries (**"***Any disability caused by medical management that prolonged the hospital stay by at least one day or persisted beyond the patient's release from hospital***" Brennan TA, Leape LL, Laird N, et al. *Incidents of adverse events and negligence in hospitalised patients: results of the Harvard Medical Practice Study I*. New England Journal of Medicine, 1991; 324: 370-376 and Leape LL, Brennan TA, Laird N, et al. *The nature of adverse events in hospitalised patients: results of the Harvard Medical Practice Study II*. New England Journal of Medicine, 1991; 324: 377-384.)" managed by CONSAP ("Concessionaria Servizi Assicurativi Pubblici" at: http://www.consap.it) a Society Dealer of Public Insurance Services with the aim of providing insurance protection when hospitals do not sign a contract according to the prescribed modalities, or if the chosen Insurance Company has been compulsory liquidated prior to the damage compensation (Articles 6, 7 and 8).

Therefore, the "**draft**" of the ***Italian National Federation of Colleges of Medical Doctors and Dentists***, and in particular the establishment of the "Guarantee Fund", coherently with the proposal of the "State-Regions Conference" dated 20/3/2008 concerning clinical risk management and patient safety, would clearly encourage providers to develop a "complementary" management of the clinical risk (and therefore of the patient safety) and to promote programs designed to decrease the number of claims and improving the quality of health care through a specific model of national governance and "risk manager" in both public and private hospitals thus attributing the "National Agency for Regional Health Services" ("AGE.NA.S.") the main functions of: a) Monitoring of Best Practices for Patient Safety and b) National Observatory on Claims and Insurance Policies. However, the same issues included in the draft bill have already been proposed through other bills on the modernization of the National Health Service that were prematurely blocked in 2004 (Draft Bill so-called "Tomassini" 108-01 on "New rules on liability of health professionals") and 2007 (Draft Bill so-called "Turco" 1920-07 on "Quality and safety measures for the National Health Service") due to government re-election. At the moment, parliamentary debate of the sixteenth legislature (of the 34 Draft Bills actually under discussion at the "***Senate Commission on Health and Hygiene***", only 7 deal with the problem of clinical risk management together with the liability in health care and compensation of patients injured by medical practice).

The final law approval on professional liability reform would balance the compensation needs of the victims with a deflation of malpractice litigation between patients, doctors and hospitals. This would decrease social scandals for every case of medical malpractice. It would also decrease devastating reflections on professional behaviors such as limiting and destabilizing the decision-making power of health professionals condemned although they had observed clinical care guidelines and recommendations (see Court of Cassation, 4th Penal Section, 02/03/2011, n. 8254), or limiting care for patients believed to be litigious, keeping more detailed medical records, referring more patients to other physicians or surgeons, and increasing the use of tests and procedures, in other words applying the so-called "defensive medicine" [7]. The consequence would be overburden of the National Health Service of alarming and unnecessary economic (and social) costs.

The professional malpractice law should allow the recovery of a system of rules on professional liability in health care (and related insurance policies) based on guarantees about fault criteria, just like in France that legislated a system of compulsory insurance (***Loi no 2002-303 du 4 mars 2002 relative aux droits des malades et à al qualité du système de santè***, in J.O., no. 54, 5 March 2002, 4118). This should be true especially for those cases characterized by high severity of iatrogenic damage (incidental or even accidental), setting up a guarantee fund dependent upon "Social Solidarity" and managing compensation and indemnification procedures through the "Regional Commissions of Conciliation and Compensation."

## Conclusions

Times are mature for the introduction in our country of a compulsory insurance system which could absorb through "distribution of risk," malpractice litigation and its costs thus providing indemnifications in accidental cases wher it is not possible to demonstrate a lack of diligence, prudence and/or skill. Conversely, in the civil liability area, "*compensation*" and "*deterrence*" must come together in a sort of "governed balance." The original system based on a sort of "immunity" of the health professional must be left behind without overflowing into a regime (like the actual one) characterized by an almost constant liability [8].

## Competing interests

The authors declare that they have no competing interests.

## Authors' contributions

SS have made substantial contributions to acquisition, analysis and interpretation of data and has been involved in drafting the manuscript; AA has been involved in drafting the manuscript and revising it critically for important intellectual content; CC has given final approval of the version to be published; PG has made substantial contributions to analysis and interpretation of data and has been involved in drafting the manuscript and revising it critically for important intellectual content. All authors read and approved the final manuscript.
